# Cholinergic Antagonists and Behavioral Disturbances in Neurodegenerative Diseases

**DOI:** 10.3390/ijms24086921

**Published:** 2023-04-07

**Authors:** Rachid Mahmoudi, Jean Luc Novella, Sarah Laurent-Badr, Sarah Boulahrouz, David Tran, Isabella Morrone, Yacine Jaïdi

**Affiliations:** 1 Department of Geriatric and Internal Medicine, Reims University Hospitals, Maison Blanche Hospital, 51092 Reims, France; 2 UR 3797 Vieillissement, Fragilité (VieFra), Faculty of Medicine, University of Reims Champagne-Ardenne, 51687 Reims, France; 3 Cognition Health and Society Laboratory (C2S-EA 6291), Faculty of Medicine, University of Reims Champagne-Ardenne, 51687 Reims, France

**Keywords:** cholinergic antagonists, anticholinergic burden, BPSD, neurocognitive disorders, dementia, aging, frailty

## Abstract

Cholinergic antagonists interfere with synaptic transmission in the central nervous system and are involved in pathological processes in patients with neurocognitive disorders (NCD), such as behavioral and psychological symptoms of dementia (BPSD). In this commentary, we will briefly review the current knowledge on the impact of cholinergic burden on BPSD in persons with NCD, including the main pathophysiological mechanisms. Given the lack of clear consensus regarding symptomatic management of BPSD, special attention must be paid to this preventable, iatrogenic condition in patients with NCD, and de-prescription of cholinergic antagonists should be considered in patients with BPSD.

## 1. Background

Acetylcholine (ACh) is the main neurotransmitter of the central nervous system. It was the first neurotransmitter to be discovered, and the scientists who first described it, Henry Dale and Otto Loewi, were awarded the Nobel Prize for the Physiology and Medicine for their discovery [1]. A cholinergic deficit in Alzheimer’s disease (AD) was described for the first time by several groups simultaneously, namely Davies and Maloney [2], Perry et al. [3] and Bowen et al. [4,5]. The cholinergic deficit observed during AD is thought to be partially linked to loss of neurons at the level of the central cholinergic nuclei; especially the nucleus basalis of Meynert [6,7]. The cholinergic hypothesis is one of the explanations for the cognitive decline observed in AD [8,9].

ACh binds to pre- or postsynaptic receptors of two different classes. The first class of receptors is composed of ligand-gated cation channels, termed nicotinic ACh receptors (nAChR). nAChR have been found in various areas of the cerebral cortex, notably the hippocampal regions, the substantia nigra, and the ventral tegmental area [10]. nAChR are homo- or hetero-pentameric transmembrane proteins comprising five subunits. The most commonly found representatives in the central nervous system are the α7 and α4β2 receptors [11,12]. Nicotinic receptors are located both pre- and post-synaptically. In the pre-synaptic context, they promote the release of glutamate destined for dopaminergic neurons [13]. At the level of the GABAergic neurons, nAChR are located post-synaptically [14]. The second class of ACh receptors, termed muscarinic receptors, signal via heterotrimeric GTP binding proteins (G proteins). Muscarinic receptors are classified into five main subtypes, namely M1, M2, M3, M4, and M5. M1 receptors are predominant, and account for 35 to 60% of all muscarinic receptors in the human brain [15]. M2 receptors are present in the presynaptic neurons of the thalamic region, the nucleus basalis of Meynert, and limbic structures such as the hippocampus and amygdala. M3 and M4 receptors are more rarely found in the brain; M3 receptors are mainly located in the hippocampus and striatum, and are implicated in the regulation of insulin secretion [16]. In a murine model, M4 receptors are inhibitory autoreceptors in glutamatergic neurons, and they regulate the metabolism of dopamine [17]. It is thought that M5 receptors are expressed at low levels in the brain [18].

The cholinergic deficit that is present from the early stages in certain neurocognitive disorders (NCD) can be aggravated iatrogenically by medical therapy. Medications with anticholinergic properties are reversible and competitive inhibitors of ACh receptors. Cholinergic antagonists act by blocking nicotinic or muscarinic receptors in the central and peripheral nervous system. They prevent ACh from binding to its dedicated receptors, thereby reducing cholinergic transmission. The majority of drugs with anticholinergic properties are not receptor-specific. Consequently, they may act on either type of the receptor, be it muscarinic or nicotinic.

The term “anticholinergic burden” (AB) refers to the cumulative effect of taking one or more medications with anticholinergic properties [19,20]. Often, the AB is the result of taking several drugs with low anticholinergic load or activity. For some medications, the anticholinergic properties are known to prescribers, and indeed constitute the desired effect, such as treatments for stress urinary incontinence. Conversely, for other medications, the anticholinergic effects maybe unknown and harmful [19,20], and may be responsible for falls, cognitive and functional decline, and delirium.

Drugs with anticholinergic effects refer to drugs that bind exclusively to muscarinic receptors (M1, M2, M3, M4, and M5). The antagonism of these receptors corresponds to their primary mechanism of action (e.g., oxybutynin, trihexyphenidyl, and ipratropium bromide), and to drugs whose anticholinergic activity is not connected with their primary therapeutic purpose and mechanism of action (e.g., antidepressants, antipsychotics, and antihistamines) [21]. Blockades of nicotinic receptor sites attributed to these drugs is negligible [22].

Most drugs with anticholinergic activity are nonselective for receptor binding and are not tissue-selective. The distribution of muscarinic receptors across many physiological systems leads to a wide range of peripheral (e.g., dry mouth, dry eyes, urinary retention, constipation, and tachycardia) and central (e.g., cognitive impairment, delirium, and confusion) adverse effects. Affinity for muscarinic receptors varies from one therapeutic class to another, and from one molecule to another within the same therapeutic class [23].

In a study by Golds et al. [24], the binding of certain antidepressants to Ach muscarinic receptors in rat brain was investigated. Most of atypical antidepressant drugs tested in this radio-ligand binding assay were muscarinic receptor antagonists. When the dissociation constants (Kd) for all the drugs tested were compared, they were found to fall approximately into different groups: Amitryptiline with the lowest Kd and therefore highest affinity of the drugs tested for the muscarinic receptor; drugs with dissociation constants in the 1 × 10^−7^ M to 7 × 10^−7^ M range: desipramine, doxepin, imipramine, maprotiline, mianserin. These drugs were 5 to 20 times less potent than amitryptiline for binding to the muscarinic receptor.

Measurement of anticholinergic activity is based on invasive techniques, either via blood tests [25], or the cerebrospinal fluid [26,27]. Non-invasive estimation of the AB can be performed using tools such as scales or indices [28], but the major drawback of this approach is the heterogeneity between instruments in terms of prediction of outcomes [29]. Given the different properties of the AB estimation tools (expert agreement, correlation with measures of serum anticholinergic activity, cross-sectional design of princeps studies), the scales are heterogeneous between them [30]. A drug can have an AB rated differently depending on the tool used as having weak or strong anticholinergic properties. Given this heterogeneity, it is difficult for the clinician to favor one scale over another. However, the choice of a scale by the clinician and/or researcher must be made according to the outcomes of interest: risk of fall, delirium, cognitive and functional decline, rehospitalization, or death [31,32,33,34,35,36,37,38].

Beyond its impact on cognitive function, the cholinergic deficit in NCD is thought to be implicated in certain behavioral and psychological symptoms of dementia (BPSD) [39,40,41]. These include notably irritability, depression, agitation, psychosis, sleep disorders, anxiety, apathy, dysphoria, aberrant motor behavior, hallucinations, and delusions [42]. BPSD affect 90% of subjects with AD, and are also common in other forms of NCD [43]. They are a frequent cause of hospitalization [44] and are associated with an increased risk of nursing home entry [45], caregiver burnout [46], and more rapid progression of NCD [47]. There is currently no consensus regarding the medical management of BPSD, and non-drug approaches should be preferred [48].

In this article, we review the established relations between exposure to anticholinergic drugs and the onset or persistence of BPSD during NCD. We outline the main pathophysiological mechanisms, and highlight the key approaches to therapeutic management, both current and future. 

## 2. Anticholinergic Burden and Behavioral Disturbances in Neurodegenerative Diseases

### 2.1. Alzheimer’s Disease

AD is the leading cause of NCD worldwide. It is characterized neuropathologically by the extracellular accumulation of amyloid-beta protein [49] and by the intracellular accumulation oh hyperphosphorylated tau protein in neurofibrillary tangles [50]. In addition to the cognitive decline occurring during the disease course, AD patients frequently experience BPSD. The most common BPSD among patients with AD are agitation [51]; delusions, apathy, hallucinations [52]; depression [53]; anxiety [54] and aberrant motor behavior [55].

In a cross-sectional study with 473 patients mostly with AD, BPSD were more frequent and more severe in patients with a higher anticholinergic burden, as assessed by the Anticholinergic Cognitive Burden (ACB) [56] and Han’s score (Han et al. JAGS 2008). Overall, the more the number of anticholinergic properties increased, the more there was a negative impact on the NPI score. However, the types of BPSD were not specified in this publication [57]. 

In a longitudinal study with 230 patients with probable AD, exposure to anticholinergic drugs was associated with an increased risk of psychosis [58]. In this study, the five most frequently prescribed anticholinergic drugs included two psychotropic agents (olanzapine and amitriptyline) and three cardiovascular drugs (warfarin, digoxin, and furosemide). 

In a study investigating the relationship between serum anticholinergic activity (SAA) and the severity of clinical symptoms of AD patients, BPSD, and particularly items of paranoid and delusional ideation and hallucinations, were found to be more frequent in those with positive SAA [59]. The authors postulated that there may be a certain level of endogenous SAA acting as a predisposing factor to BPSD among patients with AD, and that this could be aggravated by the additional anticholinergic burden deriving from prescribed drugs. In this study, most of the cholinergic antagonists to which patients with NCD were exposed belonged to the pharmacological class of psychotropic drugs.

Sunderland et al. showed that certain cholinergic antagonists (notably, intravenous scopolamine in their study) caused significant behavioral changes in patients with AD compared to age- and sex-matched controls on five domains of the Brief Psychiatric Rating Scale, namely thought disorder, hostility, anxiety/depression, mania, and activation [60].

In a sample of 125 elderly patients with NCD (mainly AD), reducing the anticholinergic burden by at least 20% yielded a significant decrease in the frequency × severity scores of the Neuropsychiatric Inventory–Nursing Home scale, and reduced the impact on the caregivers’ workload [61]. In a larger sample of 147 patients, the same group reported that using the Anticholinergic Drug Scale [62], a reduction of 2 points in patients with moderate-intensity BPSD was associated with a clinically significant reduction in the frequency and severity of symptoms, while a reduction of 3 points yielded a clinically significant improvement in the occupational disruptiveness score [63].

A prospective study by Liu et al. failed to establish a relationship between exposure to anticholinergic burden, and the occurrence of BPSD [64]. However, contrary to previous publications, BPSD were not evaluated using a validated instrument in this study, such as the Neuropsychiatric Inventory, but rather using an indirect estimation of BPSD via the prescription of antipsychotics, antidepressants, and sedative-hypnotics of estimated treatment duration ≥30 days during follow-up. Despite an average follow-up of 15 years in this cohort, this measurement bias hampers the interpretation of the findings.

In a study of 9 patients with AD who were taking medication for urinary incontinence, Jewart et al. reported that psychiatric symptoms, as measured by the NPI completed by the caregiver, did not differ between patients who were on medication and those were not [65]. Further, serum anticholinergic activity was not significantly associated with the Memory and Behavior Checklist score. However, the major limitation of this study is the small sample size, which limits the statistical power.

Accordingly, it is nowadays clear that exposure to an anticholinergic burden leads to more frequent BPSD among patients with AD, but conversely, reducing the anticholinergic burden makes it possible to attenuate BPSD in samples of subjects presenting predominantly AD. It therefore seems logical to assume that anticholinergic drugs may be implicated in the genesis and/or persistence of BPSD in AD.

These conclusions are based on the cholinergic hypothesis previously proposed by several authors [8,9]. Cummings and Kaufer [66] suggested that the cholinergic deficit was implicated in the genesis and persistence of BPSD such as delusions, depression, agitation, and apathy.

In a post-mortem study of 46 patients with NCD, of whom 36 had AD, Minger et al. showed that compared to 32 controls, cholinergic deficit in AD was associated with BPSD such as aggressive behavior and depression [67]. Similarly, Garcia-Alloza et al. investigated an imbalance between the cholinergic and serotonergic systems in a series of 22 autopsies from AD patients who had undergone prospective follow-up for cognitive impairment and BPSD prior to death. They reported that decreases in cholinergic function correlated with aggressive behavior [68].

In addition to a deficit of production of ACh due to loss of neurons at the level of the central cholinergic nuclei, genetic variation in the α7 nicotinic acetylcholine receptor gene has been shown to be associated with delusions in AD [69]. The specific implication of nicotinic receptors in the occurrence of delusions during NCD is garnering increasing attention [70]. Moreover, the blockage of these receptors by anticholinergics is thought to be involved in the onset of BPSD in patients with AD, including apathy, hallucinations, delusions, and disinhibition [66].

In patients with AD, Lai et al. used radioligand binding assays to investigate the metabolic anomalies and the density of muscarinic receptors in the frontal and temporal cortex, and correlate neurochemical findings with the presence of BPSD, notably delusions and hallucination [71]. Indeed, it is believed that the cholinergic deficit is most pronounced in the frontal and temporal cortex, and that there is increased M2 receptor binding. Moreover, in this study, authors found that M2 receptor density was significantly increased in the frontal cortex of patients with AD with hallucination and in the temporal cortex of those with delusions. These findings should be interpreted in perspective with those from an autopsy study by Wang et al., who showed a statistically significant decrease in messenger RNA (mRNA) encoding for the M1 receptor in the temporal and occipital cortex, with no change in other regions. There was also no change in the level of mRNA encoding the M2, M3, or M4 receptors in any of the brain regions studied [72]. Finally, another post-mortem study on brain specimens from patients with AD and significant psychotic symptoms, showed a loss of non-M2 muscarinic receptors in the orbitofrontal cortex, independently of the effects of dementia severity [73].

### 2.2. Lewy Body Dementia/Parkinson’s Disease Related Dementia

Synucleinopathies comprise several NCD characterized by the accumulation of aggregated forms of the α-synuclein in both neuronal and non-neuronal cells of the brain [74]. Lewy body diseases are represented by the accumulation of aggregated α-synuclein into Lewy bodies within vulnerable neurons, and Lewy neurites in neuronal processes. The Lewy body diseases comprise mainly dementia with Lewy bodies (LBD) and Parkinson’s disease (PD)-related dementia. The most common BPSD among patients with LBD are anxiety, depression, apathy, agitation, sleep disorders, and psychosis [75].

Few studies to date have investigated the impact of exposure to anticholinergics on BPSD in NCD with Lewy bodies. In a study by Jaïdi et al., five subjects with LBD were included, and a reduction in the anticholinergic burden made it possible to reduce BPSD in these subjects [61,63]. However, the very small number of subjects precludes any generalization of these results. Nevertheless, it would appear that in patients with Lewy body NCD, exposure to anticholinergics may aggravate BPSD.

Indeed, in subjects with PD (another form of synucleinopathy), there is a more marked loss of neurons in the central cholinergic nuclei than in AD, suggesting that the cholinergic deficit plays a more important role in NCD with Lewy bodies than in AD [6,7]. The cholinergic deficit is also associated with hallucinations in NCD with Lewy bodies, as a corollary of the neurodegeneration of a central cholinergic neuron, the pedunculopontine tegmental nucleus [76].

Hori et al. purported that the onset of visual hallucinations and delusions occurs earlier in NCD with Lewy bodies, because initiation of endogenous anticholinergic activity occurs earlier than in AD, under the influence of hormonal, inflammatory, and neurovegetative factors [77].

As in AD, the onset of visual hallucinations in LBD is associated with reduction in ACh-related activity in the temporal and parietal cortex [78]. In addition, there is a reduction in choline acetyl transferase (ChAT) activity in LBD, as well as an increase in M2 and M4 receptor binding [79].

According to the results of a study using in vivo SPECT imaging of muscarinic receptors using (R,R) ^123^I-QNB in patients with LBD or PDD, there is a significant increase in the binding of ^123^I-QNB in the occipital lobe (the left lobe in DLB, and the right lobe in PDD). (R,R) ^123^I-QNB is a ligand with selectivity for the M1 and M4 receptor subtypes. This mechanism appears to be involved in the occurrence of visual hallucinations and visuo-spatial impairment [80].

Moreover, it has been shown that there is a more marked reduction in the M1 subtype of muscarinic receptors in LBD than in AD, and blockade of these receptors is thought to be associated with complex visual hallucinations in NCD with Lewy bodies [81,82,83]. Shiozaki et al. demonstrated in necropsied brains from a series of 7 patients with LBD and 11 patients with AD that there was a higher proportion of M1 receptors in LBD patients, and M2 receptors in AD patients. The authors purported that the deregulation of the cholinergic system differs between the two diseases, and they further hypothesized that presynaptic cholinergic depletion due to a more marked loss of neurons in the central cholinergic nuclei than in AD causes the upregulation of M1 receptors in DLB [81].

### 2.3. Frontotemporal Dementia

Frontotemporal dementia refers to a group of NCD that encompasses progressive dysfunction in executive functioning, behavior disturbances, and language. As its name indicates, it is a cluster of syndromes that results from the degeneration of the frontal ant temporal lobes, and is subdivided into two categories that are unique with respect to their predominating presentations: behavioral subtype (bvFTD) and language subtype [84]. The most common BPSD found during the course of the bvFTD are disinhibition, apathy, loss of empathy or sympathy and preservative, stereotyped or compulsive behaviors [85].

To the best of our knowledge, no study to date has investigated the impact of anticholinergic burden on BPSD in the setting of bvFTD. Compared to AD, the cholinergic system is relatively well preserved in FTD. However, autopsy studies in subjects with FTD have shown a reduction in muscarinic receptors [86,87]. This suggests that exposure to anticholinergics may be harmful in patients with FTD.

### 2.4. Vascular Dementia

Vascular dementia (VaD) refers to a decline in mental ability, severe enough to interfere with daily life. The Vascular Impairment of Cognition Classification Consensus Study defines VaD as a clinical deficit in at least one cognitive domain that is of sufficient severity to cause a severe disruption of (instrumental) activities in daily living [88]. According to this classification, VaD is classified into four major subtypes: post-stroke dementia (defined as a dementia within 6 months after a stroke); subcortical ischemic VaD; multi-infarct (cortical) dementia; and mixed dementia. VaD is associated with irritability, apathy, depression, sleep/night-time behaviors, and agitation [89].

Again, no study to date has specifically evaluated the effects of anticholinergic burden on BPSD in a sufficiently large sample of patients with VaD. Of note, in the studies published by Jaïdi et al., 13.6% of subjects included had VaD and almost 25% had mixed NCD (AD and VaD) [61,63]. It was shown in these patients that a reduction in the anticholinergic burden led to an improvement in BPSD symptoms.

In VaD, cholinergic deficits may result from ischemia of the central cholinergic nuclei, which are very sensitive to ischemia since they are irrigated by perforating arteries. The cholinergic deficit observed in VaD may also arise from white matter lesions that alter cholinergic input to the cortex [90,91]. Accordingly, in patients with VaD, and independently of any AD-like pathology, cholinergic activity is diminished [27,92].

**Figure 1:**
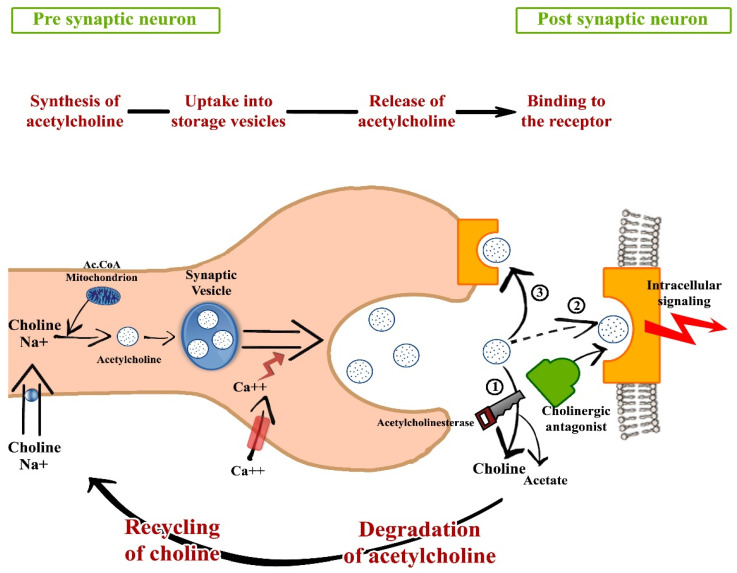
Cholinergic synapse. Mechanisms of action of cholinergic antagonists. 1. Acetylcholinesterase hydrolyzes ACh to inactive choline and acetate. 2. Cholinergic antagonists compete with Ach for binding to post-synaptic receptors. Non-competitive antagonism: the cholinergic agonist and antagonist can bind simultaneously, but the binding of the cholinergic antagonist reduces or inhibits the action of the agonist. Competitive antagonism: binding of the antagonist to the receptor prevents the binding of the agonist to that same receptor. 3.Presynaptic muscarinic autoreceptor performing a negative feedback loop in signal transduction.

Table 1 presents the main clinical studies that have evaluated the relationship between BPSD and AB according to the type of NCD and Figure 1 shows the mechanism of action of cholinergic antagonists at the cholinergic synapse.

Alzheimer’s disease: the cholinergic deficit is most pronounced in the frontal and temporal cortex, and there is an increase in binding to the M2 receptor [67]. There is a loss of non-M2 muscarinic receptors in the orbitofrontal cortex, independently of the effects of dementia severity in AD patients with psychotic symptoms [69].

Lewy body dementia/Parkinson’s disease related dementia: There is increased binding in the occipital lobe with selectivity for the M1 and M4 receptor subtypes, and this mechanism appears to be involved in the occurrence of visual hallucinations and visuo-spatial impairment [76]. Furthermore, there is an increased binding in the occipital lobe with selectivity for the M1 and M4 receptor subtypes, and this mechanism appears to be involved in the occurrence of visual hallucinations and visuo-spatial impairment [77,78,79].

Fronto-temporal dementia and Vascular dementia: There are no studies investigating the behavioral outcomes of muscarnic receptor binding in FTD and VaD.

## 3. How Can We Lessen the Impact of AB on BPSD?

The iatrogenic effects arising from the anticholinergic burden are largely avoidable. Although the anticholinergic properties of some drugs are unknown or occult, they can be detected more easily by the systematic use of scales to estimate anticholinergic burden [28]. These scales present the notable advantage of being non-invasive. However, there is a certain degree of heterogeneity between the existing instruments [29]. Thus, the prescriber should assess the anticholinergic burden when initiating a treatment, or during periodic medication reviews (e.g., at the occasion of a hospitalization or admission to a nursing home). Within a deprescribing framework, in addition to the leading role of the prescriber, the pharmacist can also be a key contributor to reducing the anticholinergic burden (via such approaches as medication review, academic detailing, education of healthcare practitioners, and computerized clinical decision support systems), especially among older populations, as shown in several therapeutic trials [93]. Deprescription of anticholinergics seems to be feasible, even in a population of frail geriatric patients [94]. To be profitable, this strategy would be desirable to specifically target psychotropic drugs given the prevalence of their prescription in the elderly population on the one hand [95,96], and their significant anticholinergic burden on the other hand [97,98].

As far as possible, a cholinergic antagonist whose indication is indisputable should be replaced by another molecule of the same class using the anticholinergic load estimation scales. The Anticholinergic Risk Scale [99] seems to date to have the best correlation with pharmacokinetic parameters [23]. Moreover, in a situation of overuse, cholinergic antagonist cannot be renewed.

A trial is currently underway to compare the effectiveness of different types of reduction interventions (e.g., pharmacist-led versus GP-led; educational versus audit and feedback) in reducing overall anticholinergic burden; to establish optimal duration of anticholinergic reduction interventions; sustainability and lessons learned for upscaling; and to compare results according to differing anticholinergic scales used in medication reduction intervention trials [100].

Despite the prevalence of BPSD and its hazards in patients with NCD, there has not yet been medication that is clearly effective and approved by the Food and Drug Administration or the French High Health Authority. Again, and in the absence of approved therapy targeting BPSD with a satisfying benefit/risk balance, it seems essential to pay a special attention not to worsen a cholinergic deficit (suspected or proven). Every practitioner should avoid as far as possible cholinergic antagonists in patients with NCD whether they present with BPSD or not.

Cholinesterase inhibitors (CI) have shown to yield a clinically significant, albeit modest improvement in BPSD in both AD and NCD with Lewy bodies [101,102]. Nevertheless, in the case of FTD, CI have been shown to be inefficacious at best in improving BPSD [103], and may even aggravate symptoms [104]. Furthermore, memantine alone or in combination with CIs participates in the improvement of BPSD with a different effect size from one study to another [31,105,106,107,108].

Finally, muscarinic ACh receptors seem to be a promising therapeutic target in BPSD, in which a cholinergic deficit is implicated, particularly in AD and NCD with Lewy bodies. Indeed, subtype-selective allosteric modulators for muscarinic ACh receptors (coupled with G protein) have shown promising therapeutic potential and may yield a benefit in the management of BPSD [109,110].

Table 2 illustrates the different types of ACh receptors and therapeutic targets of potential interest.

## 4. Further Considerations/Conclusions

This brief review illustrates that to date, few clinical studies have investigated specifically the impact of the AB in BPSD. There is a strong presumption based on the imbalance between neurotransmitters, especially ACh, and receptor dysfunction, but an actual deleterious effect of anticholinergics on BPSD in NCDs other than AD has never been clearly demonstrated. Nevertheless, despite the current lack of robust evidence, special attention should be paid to avoid medication-induced iatrogenic effects, notably due to anticholinergic drugs, in patients presenting with NCD. This could be achieved by practicing a “zero exposure” policy in patients with NCD, based on the “safety-first” principle. Other prospective clinical studies are warranted to evaluate the impact of the anticholinergic burden and the effect of deprescription of anticholinergics on BPSD.

## Figures and Tables

**Table 1:** Summary of the main studies that have investigated the association between BPSD and AB, according to the type of NCD.

	Author, Year	Type of Study	Anticholinergic Burden Assessment Tool, if Used	Outcome
Alzheimer’s disease	Dauphinot, 2017 [57]	Cross-sectional	ADS, ACB, ARS, Chew’s score, Han’s score	Higher NPI was associated with the Han’s score
	Cancelli, 2008 [58]	Cross-sectional	none	AD patients exposed to AB were more likely to have psychosis
	Hori, 2011 [59]	Cross-sectional	SAA	Elevated SAA was linked to higher BEHAVE-AD *
	Sunderland, 1987 [60]	Cross-sectional	none	After IV scopolamine 0.25 mg, AD patients showed anxiety, depression, and agitation
	Jaïdi, 2018 [61]	Cross-sectional	ADS, ACB, ARS	Reducing AB by at least 20% enabled a significant decrease in BPSD
	Liu, 2020 [64]		ACB	Anticholinergic usage was not associated with BPSD
	Jewart, 2005 [65]	Cross-sectional	SAA	No significant relationship was found
Lewy body dementia Parkinson Disease dementia	Jaïdi, 2018 [61] *	Cross-sectional	ADS, ACB, ARS	Reducing AB by at least 20% enabled a significant decrease in BPSD
Fronto-temporal dementia	x	x	x	x
Vascular dementia	Jaïdi, 2018 [61] ***	Cross-sectional		Reducing AB by at least 20% enabled a significant decrease in BPSD

ADS: Anticholinergic Drug Scale. ACB: anticholinergic cognitive burden. ARS: Anticholinergic Risk Scale. NPI: NeuriPscyhiatric Inventory. AD: Alzheimer’s disease. AB: anticholinergic burden. SAA: serum anticholinergic activity. BEHAVE-AD: Behavioral Pathology in Alzheimer’s Disease Rating Scale. AB: anticholinergic burden. * In particular paranoid and delusional ideations and diurnal rhythm disturbances. *** 13.6% of subjects included had VaD and almost 25% had mixed NCD (AD and VaD).

**Table 2:** Types of ACh receptors and potential therapeutic targets.

Receptor	Central Location	Mechanism of Action	Involvement in Cognition/BPSD	Therapeutic Target
**Muscarinic**				
**M**	Cortex, hippocampus, striatum, and thalamus, post-synaptic [111]	Coupled with protein G_q_ (activates phospholipase C)	Memory Learning [112]	M1 agonists: -Xanomeline LY593093 improved cognitive function and reduced BPSD such as agitation, delusions, and hallucinations in patients with AD [113] -GSK1034702 is an allosteric M1 agonist in clinical development for the treatment of cognitive disorders in AD (NCT00743405) [114] -Heptares announced positive phase I results with the molecule HTL9936 (NCT02291783) Positive allosteric M1 modulators: -Benzyl quinolone carboxylic acid, BQCA, improved memory performance in patients with AD [115] -PF-06767832: poor gastro-intestinal and cardiovascular tolerance [116]
**M2**	Brainstem, thalamus, cortex, hippocampus and striatum [117]	Coupled with protein G_i/o_ (inhibits adenylyl cyclase and modules ion channels)	Memory Learning Delusions Hallucinations [71]	Selective and active M2 antagonists M2 currently being optimized [118] in view of their poor tolerance [119]
**M3**	Cortex and hippocampus [111]	Coupled with protein G_q_ (activates phospholipase C)	Memory Learning [120]	
**M4**	Striatum, cortex, Hippocampus [111]	Coupled with protein G_i/o_ (inhibits adenylyl cyclase and modules ion channels)	Regulation of ACh turnover [121,122]	M4 Agonists: -Heptares announced the development of selective M4 and mixed M1/M4 agonists Positive allosteric M4 Modulators: -LY2033298 [123] but has not yet entered clinical development [116]
**M5**	Locus niger [111]	Coupled with protein G_q_ (activates phospholipase C)	Delusions Hallucinations [73]	
**Nicotinic**	Glutamateric and dopaminergic neurons; GABAergic and cholinergic interneurons [124,125]	Ionotropic	Delusions [70] Hallucinations [126]	-AR-R17779 improved social recognition by activation of α7 nicotinic receptor [127] -N-[128]oct-3-yl-7-[128]-1-benzofuran-2-carboxamide improves working memory and recognition [128]

BPSD: behavioral and psychological symptoms of dementia. AD: Alzheimer’s disease.

## Data Availability

Not applicable.
